# OSMAC Strategy Integrated with Molecular Networking for Accessing Griseofulvin Derivatives from Endophytic Fungi of *Moquiniastrum polymorphum* (Asteraceae)

**DOI:** 10.3390/molecules26237316

**Published:** 2021-12-02

**Authors:** Victor F. Farinella, Eunizinis S. Kawafune, Marcelo M. P. Tangerina, Helori V. Domingos, Leticia V. Costa-Lotufo, Marcelo J. P. Ferreira

**Affiliations:** 1 Departamento de Botânica, Instituto de Biociências, Universidade de São Paulo, São Paulo 05508-090, SP, Brazil; victor.farinella@usp.br (V.F.F.); kawafune.nizi@gmail.com (E.S.K.); marcelotangerina@usp.br (M.M.P.T.); 2 Departamento de Farmacologia, Instituto de Ciências Biomédicas, Universidade de São Paulo, São Paulo 05508-090, SP, Brazil; hvanni@icb.usp.br (H.V.D.); costalotufo@usp.br (L.V.C.-L.)

**Keywords:** endophytes, *Gochnatia polymorpha*, compositae, griseofulvin derivatives, polyketides, dereplication, metabolomics

## Abstract

Three endophytic fungi isolated from *Moquiniastrum polymorphum* (Less.) G. Sancho (Asteraceae) were cultivated using the one strain many compounds (OSMAC) strategy to evaluate the production of griseofulvin derivatives. Extracts obtained were analyzed by HPLC–MS/MS and the chromatographic and spectrometric data used to elaborate a feature-based molecular network (FBMN) through the GNPS platform. This approach allowed the observation of differences such as medium-specific and strain-specific production of griseofulvin derivatives and variations of cytotoxic activity in most extracts. To evaluate the efficiency of the OSMAC approach allied with FBMN analysis in the prospection of compounds of biotechnological interest, griseofulvin and 7-dechlorogriseofulvin were isolated, and the relative concentrations were estimated in all culture media using HPLC–UV, allowing for the inference of the best strain–medium combinations to maximize its production. Malt extract-peptone broth and Wickerham broth media produced the highest concentrations of both secondary metabolites.

## 1. Introduction

The *Moquiniastrum* genus (Asteraceae) contains 21 species distributed in South America and 19 species endemics to Brazil [1]. *Moquiniastrum polymorphum* (Less.) G. Sancho, also known as cambará, is a medium-sized tree found in eastern Brazil, as well as Argentina, Paraguay, and Uruguay [2]. Ethnobotanical studies have reported the use of *M. polymorphum* leaves in infusions to treat sore throat [3]. Phytochemical studies showed the presence of sesquiterpene lactones, diterpenes, triterpenes and coumarins [4,5,6,7]. Within the secondary metabolites described, sesquiterpene lactones presented anti-inflammatory and antineoplastic activities in vivo [2,8]. However, studies of the endophytic microbiota have been not described for *Moquiniastrum* genus in the literature.

Endophytic fungi are diverse polyphyletic groups, and they asymptomatically inhabit healthy plant tissues [9,10]. The presence of these microorganisms has been related to benefits for the host plant, such as growth promotion and resistance against pathogens, drought, and herbivory [10,11,12]. Furthermore, endophytic fungi can produce a wide range of secondary metabolites, many of them with activity against various diseases [13,14]. Within the endophytic microorganisms isolated from Asteraceae species, many fungi have been shown to produce bioactive secondary metabolites, such as chaetoglobosin B [15] and aphidicolin [16] with anti-tumoral activity, and terrenolide S with antileishmanial activity [17]. However, the expression of many biosynthetic gene clusters (BGCs) of secondary metabolites can remain undetected in standard laboratory conditions [14,18,19]. Thus, several methods have been developed to activate these cryptic gene clusters and to augment the fungi chemodiversity.

Systematic variation of culture conditions, also known as the one strain many compounds (OSMAC) strategy, has been successfully applied to access the diversity of secondary metabolites in fungi, leading to the discovery of new and bioactive metabolites. Cultivation of *Sphaeropsidales* sp. using three culture media resulted in six new compounds [20]. The endophytic fungus *Bulgaria inquinans* growth in two culture media produced 14 new compounds, including one butyrolactone with high cytotoxic activity [21]. Although the efficiency of the OSMAC strategy for diversifying the secondary metabolite repertory of one strain is very well documented in literature, this approach is especially interesting for the prospection of compounds of biotechnological interest, such as griseofulvin, an antifungal polyketide produced by several fungi species [22,23].

Griseofulvin, firstly isolated from *Penicillium griseofulvum*, was initially used to inhibit and treat superficial fungal infections [24]. It has been used widely as a drug of choice for tinea capitis [25]. Additionally, this compound has been explored for its antimitotic properties for many years [26]. Griseofulvin interferes with microtubule dynamics, which leads to retardation of organization and function of mitotic spindles, inducing apoptosis and thereby ultimately causing cell-cycle arrest in the G2M phase in human cervical cancer (HeLa) cells [27], in human promyelocytic leukemia cells (HL-60) by activation of the NF-κβ pathway [28], in human colorectal cells by downregulating myt-1 protein expression and inducing elevated cyclin BI/cdc2 kinase activity [29] and in human leukemia (K562 cells) inducing apoptosis and mitochondrial membrane potential loss [30]. Furthermore, it also enhances activation of caspases 3 and 9 [29,30]. Griseofulvin has played a significant role as an inhibitor of centrosome coalescence, inducing multipolar spindle formation in many human cancer cell lines, such as cervical (HeLa), MCF-7 (breast), SCC114 (oral), and U2OS (osteosarcoma), and causing mitotic arrest and cell death in tumor cell lines without affecting keratinocytes and diploid fibroblasts with normal centrosome content, and thus sparing healthy tissues by selectively targeting only tumor cells [31].

To date, there are no studies employing the OSMAC approach to investigate griseofulvin-producing fungi. Thus, the endophytic fungi obtained from *Moquiniastrum polymorphum* (Asteraceae) were selected based on the production of griseofulvin derivatives [23]. The main goal of this study was to assess the variation in the production of griseofulvin derivatives when the strains were submitted to different culture conditions. The main questions addressed herein were the following: (1) what is the best cultivation medium for production of these compounds?, and (2) which carbon and nitrogen sources provide better yields of the target compounds?. Furthermore, the cytotoxicity was evaluated from the obtained extracts.

## 2. Results and Discussion

Eighty-six strains of endophytic fungi were recovered from *Moquiniastrum polymorphum* and codified as MPO574 to MPO660. These strains were cultured on potato dextrose agar (PDA) at 25 °C for 28 days and, subsequently, extracted with ethyl acetate. The crude extracts were obtained and analyzed by HPLC–DAD-UV-MS/MS as a preliminary study of metabolic diversity. Each strain’s griseofulvin producers were detected from the chromatogram of the extracted ion of compound ([M + H]^+^ = 353.07). As a result, three strains (MPO611, MPO620, and MPO658) were selected for further study.

According to molecular data of the ITS region, a barcode region for identification of filamentous fungi [32], the three strains MPO611, MPO620, and MPO658 were identified respectively as *Phomopsis* sp., *Hypoxylon* sp., and *Aspergillus* sp. (GenBank accession numbers: OL545265, OL545266 and OL545267, respectively). Species of these three genera occur as endophytic, phytopathogenic and saprophytic fungi [33,34]. These genera are known to produce bioactive compounds such as cytochalasins, phomopsins, cytosporones, indole diterpenes and triterpenes [34,35,36,37,38,39] as well as griseofulvin derivatives [23].

**Figure 1:**
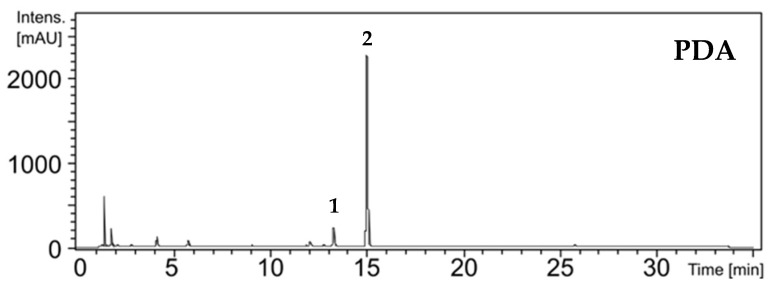
Example of chromatogram obtained from PDA crude extract of MPO620 strain (*Hypoxylon* sp.). Compounds **1** and **2** were identified as 7-dechlorogriseofulvin and griseofulvin, respectively.

PDA extracts of the MPO611 (*Phomopsis* sp.), MPO620 (*Hypoxylon* sp.) and MPO658 (*Aspergillus* sp.) strains presented two major compounds with retention times at 13.3 and 15.0 min (Figure 1 and Figure S1). Both compounds were isolated, by semi-preparative HPLC, from the ethyl acetate extracts of the three producing strains when cultivated in malt-extract peptone (MEP) and Wickerham (WKM) culture media. These media had the highest yields of crude extracts as well as the highest relative percentage of compounds. Consequently, parts of these crude extracts were pooled and selected for chromatographic separation. This procedure yielded 3.6 mg and 3.5 mg of compounds and their structures were identified by NMR as 7-dechlorogriseofulvin and griseofulvin, respectively (Table S1). Similar metabolic profiles for one strain cultivated in different culture media are undesirable for bioprospecting and chemodiversity studies. However, a metabolic profile with few intense compounds can be an advantage for studies aiming to optimize production of a specific compound. A notable example is the 10-fold intensification of penicillin production by *Penicillium chrysogenum* when cultured in culture media with different carbon and nitrogen sources [40].

To assess the variation in the production of griseofulvin derivatives, the three strains were submitted to six culture conditions for 28 days (see Experimental section). Subsequently, the cultures were extracted with ethyl acetate and the crude extracts were analyzed by HPLC–DAD-UV-MS/MS. To present a holistic analysis of the HPLC–MS/MS results, a comparative metabolomic approach was chosen through the generation of a feature-based molecular network (FBMN) by the GNPS platform [41]. The FBMN generated consists of mass spectrometry and chromatographic data of the three identified strains cultivated in six culture media. This molecular networking was formed by 5519 nodes, grouped in 549 clusters with 127 annotated compounds, where at least 58 clusters contained one annotated compound. Each node represented a molecular ion subdivided as a pie chart, where each culture medium was represented using one color and each group of linked nodes formed a cluster. Some clusters were formed by compounds with medium-specific production, taking into consideration the predominance of one color (Figure S2). The clustering of compounds can show the production of a specific class of secondary metabolites in one or more culture media since related nodes are clustered by matching the mass spectra of compounds [41].

**Figure 2:**
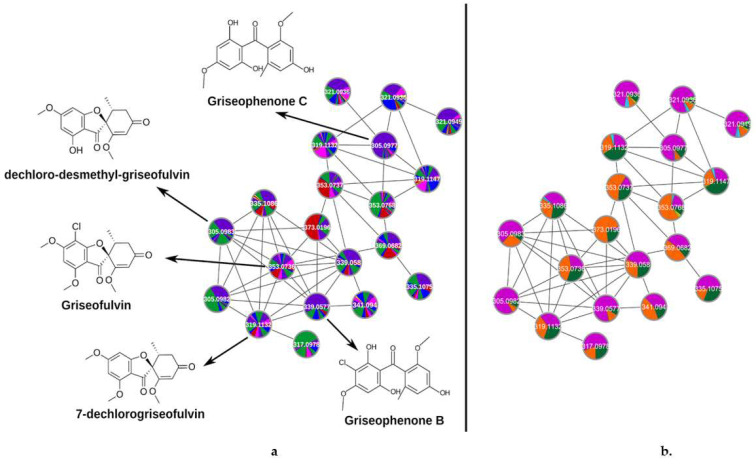
Example of clusters from the molecular network of the identified griseofulvin derivatives and of putatively new unknown metabolites. (**a**) Griseofulvin cluster with color codes discriminate nodes occurring in the extracts obtained from different culture media: blue: rice (R); green: Wickerham modified broth medium (WKM); pink: Czapek broth (CZK); purple: malt extract peptone broth (MEP): red: potato dextrose agar (PDA); and yellow: potato dextrose broth (PDB). The size of a slice is proportional to the relative abundance of a compound. Slices with the same color indicate the production of the compound by more than one strain in the referred culture medium. (**b**) Griseofulvin cluster with color codes discriminate nodes from different strains: dark pink: MPO611 strain (*Phomopsis* sp.); orange: MPO620 strain (*Hypoxylon* sp.); and dark green: MPO658 strain (*Aspergillus* sp.). Comparing both charts, it is possible to overlap the slices and find the best strain-culture medium combination by color coding for a specific node.

A comparative analysis using the same cluster organized by culture medium (Figure 2a) and by producing strain (Figure 2b) may lead to the discovery of the best strain-culture medium combination to produce a respective compound. Using two color codes side by side (by culture medium and strain), Figure 2 shows the griseofulvin cluster with their respective annotated compounds, which have previously been described as products of fungi secondary metabolism [23]. The production of each compound in different culture media is shown by the presence of slices with different colors (Figure 2a). The presence of more than one slice with the same color indicates the production of the respective compound for more than one strain in the same culture medium, as indicated in Figure 2b. A visual analysis of the colors and sizes of slices in one node can show the relative abundance of the compound produced in different culture conditions. For example, griseofulvin and 7-dechlorogriseofulvin were produced in all culture media except in PDB medium. Both compounds were produced in high numbers in MEP (purple) and WKM (green) media. Moreover, to the best of our knowledge, this is the first report of griseofulvin production by a fungus of the *Hypoxylon* genus. Griseophenones B and C are produced abundantly by the MPO611 strain in MEP medium in comparison to other strains or culture media. This strain also has the highest production of dechloro-desmethyl-griseofulvin.

To explore the quantitative aspect of FBMN analysis, all the annotated compounds from each strain–medium combination were classified with respect to their MS peak areas. Several chemical classes could be observed among the annotated compounds, such as polyketides, xanthones, diketopiperazines, and isochromenones. Within the 127 annotated compounds, griseofulvin and 7-dechlorogriseofulvin showed the highest peak areas, indicating that they were the most abundant compounds in various combinations. They were found in 14 of the 18 extracts from the three studied strains that compose the FBMN. Therefore, to evaluate the efficiency of the OSMAC approach allied with FBMN analysis in the prospection of compounds of biotechnological interest, griseofulvin and 7-dechlorogriseofulvin were quantified in the extracts.

To determine the concentration of these compounds in all strain–medium combinations indicated by the FBMN analysis, a calibration curve was elaborated and analyzed by HPLC–UV(DAD) for each compound (Figures S3 and S4). Quantification results of the ethyl acetate extracts from all culture conditions of the three strains are shown in Table S2.

When comparing the relative percentage of griseofulvin by culture media used (Table 1), MEP medium showed the highest values, except when used with the MPO620 strain (*Hypoxylon* sp.). Furthermore, the MEP and WKM media showed the highest percentage of 7-dechlorogriseofulvin. However, greater percentage differences were observed between 7-dechlorogriseofulvin strains. The highest percentages of griseofulvin (7.74%) and 7-dechlorogriseofulvin (4.54%) were achieved by the cultivation of the MPO620 strain (*Hypoxylon* sp.) in WKM medium. Brian and collaborators evaluated the best culture conditions for “curling-factor” production by *Penicillium janczewskii* [42], which was subsequently identified as griseofulvin by Grove and McGowan [43]. Two culture media were used: Czapek-Dox medium with dextrose as a carbon source and sodium nitrate as a nitrogen source, and Raulim-Thom medium with dextrose as a carbon source and ammonium salts as a nitrogen source. The griseofulvin concentration in Czapek-Dox medium (80 mg·L^−1^) was at least 200 times greater than the highest concentration observed in CZK medium (0.4 mg·L^−1^) in this study. The difference between both media is the sucrose as a carbon source in CZK medium.

The production of griseofulvin by 11 strains of *Penicillium griseofulvum* was evaluated using two culture media: YES with sucrose as a carbon source and yeast extract as a nitrogen source and Wickerham with sucrose as a carbon source and a mixture of peptone, sodium nitrate, and corn steep liquor as a nitrogen source [44]. The authors observed that Wickerham medium led to the highest observed percentage of griseofulvin (3.25% of extract dry mass), less than a half of the yield obtained by our study with the strain MPO620 (*Hypoxylon* sp.) cultivated in WKM medium (7.74%). The WKM medium used in this work differs from Wickerham in its use of micronutrients solution, its use of dextrose as a carbon source and the absence of sodium nitrate and corn steep liquor as a nitrogen source. In 1962, the British company Glaxo registered a patent describing the production of griseofulvin on a large scale by *Penicillium* sp. strains. The best concentration (14 g·L^−1^) was achieved in a culture medium with dextrose as a carbon source and a mixture of ammonium sulfate and corn steep liquor [45].

According to the available literature as well as the results obtained from this study, carbon and nitrogen sources have a significant impact on griseofulvin production. Griseofulvin and 7-dechlorogriseofulvin were poorly produced or could not be detected in poor nitrogen culture media (PDB and PDA) in the three strains, with exception of the high griseofulvin percentage in PDA culture of the MPO620 strain (*Hypoxylon* sp.). Cultivation in CZK medium also led to a lower percentage of both compounds (Table S2) when compared to culture media containing expressive nitrogen sources such as MEP and WKM (Table S2). The nature of the nitrogen source can be a key factor in producing both compounds in the studied strains since CZK has an inorganic source (nitrate) and MEP and WKM media have an organic source (peptone). This fact has been observed in literature for other fungi. For example, the use of nitrate as a nitrogen source in the cultivation of *Aspergillus* species resulted in repression of aflatoxin synthesis but enhancement of sterigmatocystin synthesis, showing a dual effect of the nitrogen source depending on the secondary metabolite [46]. However, the high griseofulvin percentage in PDA is surprising, although it is known in the literature that different physical states of the same culture medium can provide changes in metabolic production. Different culture regimes of *Penicillium* sp. strains led to specific production of aromatic polyketides in solid or liquid cultures [47]. Thus, the difference in its metabolic profile from that of the MPO620 strain can be explained by different culture states (PDA: solid and PDB: liquid) since this strain had the highest percentual in solid media (PDA and rice) in comparison to the other two strains.

Quantity and quality of nitrogen is an important factor in the production of secondary metabolites, regardless of the presence of nitrogen in the final compound [48,49]. The presented results suggest the use of dextrose or maltose as carbon source and an organic source of nitrogen as the best combination for griseofulvin and 7-dechlorogriseofulvin production by the selected strains.

The impact of the OSMAC approach was also evaluated on the cytotoxicity of extracts. The results of human colon carcinoma cell line (HCT-116) inhibition for each strain–medium combination are shown in Table 2. Rice and MEP extracts from the MPO611 (*Phomopsis* sp.), MPO620 (*Hypoxylon* sp.) and MPO658 (*Aspergillus* sp.) strains showed cell inhibition values greater than 60%. In literature, evaluation of cytotoxic activity in 26 endophytic fungi, cultured in PDB, Czapek and Wickerham media has shown a high influence of the culture medium, since five of these strains were only active when cultured in PD medium [46]. Variations in cytotoxic activity can be the result of the production of a new metabolite in a specific culture medium or the higher production of an existing bioactive metabolite. For example, cultivation of *Fusarium tricinctum* in rice medium with different supplementations resulted in an 80-fold increase of a cytotoxic compound named fusarielin J when compared to cultivation in pure rice [50]. In this study, the OSMAC approach led to a variation of cell growth inhibition from the same strain in different culture media, indicating the efficiency of the strategy for extracting bioactive metabolites. However, no correlation was observed between the cytotoxicity and the concentration of griseofulvin and 7-dechlorogriseofulvin observed in FBMN for any strain–medium combination. In fact, other compounds produced by these strains and present in the crude extracts of the different culture media interfered in the cytotoxic activity observed for the samples.

## 3. Materials and Methods

### 3.1. General Experimental Procedures

NMR spectra were recorded on a Bruker AIII 500 MHz spectrometer (Bruker-Biospin, Bremen, Germany) operating at 500.11 MHz for ^1^H NMR at the Institute of Chemistry of the University of São Paulo. Chloroform-D (Sigma-Aldrich, St. Louis, MO, USA) was used as the solvent and internal standard. HPLC-grade solvents of the trademark J.T. Baker were used for the HPLC chromatography analyses. Analytical HPLC–DAD-UV analyses were performed on an Agilent 1260 chromatograph (1260 Infinity LC system, Agilent Technologies, La Jolla, CA, USA) equipped with an ultraviolet spectrum scanning detector by arrangement of photodiodes with a 60 mm flow cell. Zorbax Eclipse plus reverse phase C18 (4.6 × 150 mm) with 3.5 μm particle diameter was used as the stationary phase and a flow rate of 1.0 mL·min^−1^ was employed for analysis on an analytical scale. For separation of compounds, the Agilent 1200 semi-preparative chromatograph system was used with a C18 Zorbax eclipse plus LC-18 column (25 cm × 10 mm) with 5 μm diameter particles and a flow rate of 4.176 mL·min^−1^. HPLC–HR-ESI-MS/MS data were obtained on an Agilent 1290 (Agilent, La Jolla, CA, USA) using a reverse phase column (Kinetex C18 2.6 µm, 150 × 2.1 mm) with a flow rate of 0.4 mL·min^−1^ and coupled to a quadrupole time-of-flight mass spectrometer (Micro-TOF QII system; Bruker Daltonics, Billerica, MA, USA) equipped with ESI operating in a positive ion mode at 18.000 FWHM of mass resolution.

### 3.2. Isolation, Culture and Extraction, Selection Criteria and Identification of Endophytic Fungi Strains

Endophytic fungi were isolated from leaves and stems of *Moquiniastrum polymorphum* (Less.) G. Sancho (syn: *Gochnatia polymorpha* (Less.) Cabrera) collected in São Paulo state, Brazil. A specimen voucher (Farinella 02) was deposited at Herbarium of University of São Paulo (SPF). All collected plant parts were washed and the surface sterilization was carried out following a previously established method [15,37]. Leaves and stems were submitted to external asepsis to ensure the elimination of dirt and epiphytic microorganisms. Initially, the plant parts were washed only with running water to remove dust, soil, and other solid waste. The parts were then dipped into beakers containing 70% ethanol (3 min), 1% hypochlorite solution (5 min) and finally 70% ethanol (1 min). The plant material was then transferred to a laminar flow (Pachane, class Pa40, vertical flow) where it was washed in two beakers with sterile water to remove residues of alcohol or hypochlorite. In a sterile environment, small fragments of approximately 3 mm^2^ were cut and placed into Petri dishes with potato dextrose agar (PDA) medium containing ampicillin (50 mg·L^−1^) to prevent bacterial growth. The cultures were monitored every day to verify the growth of endophytic fungi. Each morphologically different mycelium that grew out from the explant fragment was subcultured to a plate with PDA medium.

All cultures (86 strains codified as MPO574 to MPO660) were incubated at 25 °C for 28 days. Subsequently, the contents of the Petri dishes (mycelium plus PDA) was fragmented, transferred to an Erlenmeyer flask and extracted with ethyl acetate (50 mL) under stirring for 24 h. After filtration, solvent was removed under reduced pressure and the crude extracts were obtained. Each extract was solubilized in LC–MS-grade methanol (2 mg·mL^−1^), filtered using a 0.45 µm syringe filter and analyzed by HPLC–HR-ESI-MS/MS. The analysis was carried out with a sample injection volume of 5 µL and the column temperature was set at 40 °C. The chromatographic method used a gradient of mixtures of solvents A (0.1% acetic acid in H_2_O) and B (acetonitrile) of: 0–3 min (10–25% B); 3–10 min (25–50% B); 10–14 min (50–100% B) and 14–19 min (100% B). The mass spectra were acquired in positive ion mode in the range of 50–1200 m/z. The strains were selected by the presence of griseofulvin in the crude extracts, which was detected from the chromatogram of extracted ion of compound ([M + H]^+^ = 353.07).

The previously selected three fungi strains were identified through sequencing of ITS1-5.8S-ITS4 region of the rDNA. Each strain was cultivated in Czapek broth medium in static condition at 25 °C until a mycelial mat was formed on the surface. The mycelium was separated from the culture broth and ground using liquid nitrogen. Subsequently, genomic rDNA was extracted and purified using the Wizard^®^ Genomic DNA Purification Kit (Promega, Madison, WI, USA). The ITS regions of the fungi were amplified by PCR (Veriti 96-well Thermal cycler, Applied Biosystems) with the universal primers ITS1 (5′-TCCGTAGGTGAACCTGCGG-3′) and ITS4 (5′-TCCTCCGCTTATTGATATGC-3′). The PCR products were purified using the Wizard^®^ SV Gel and PCR Clean-Up System (Promega, Madison, WI, USA) and sequenced using Sanger’s dideoxynucleotide chain termination method in a 3730 DNA Analyzer (Thermo Fisher Scientific, Waltham, MA, USA). The obtained sequencing data were aligned and prepared with FlichTV and Bio-Rad softwares. The consensus sequence was matched using three available online databases: BLAST (NCBI), MycoBank and Bold Systems. To determine the phylogenetic distance of strains in the same family, phylogenetic trees were elaborated using the maximum parsimony method with 1000 replicates through the bootstrap test and Min-mini heuristic algorithm using MEGA7 software [51] (data not shown).

### 3.3. Fermentation and Extraction of Strains to “One Strain Many Compounds” (OSMAC) Approach and Feature-Based Molecular Networking (FBMN) Data Processing

Evaluation of griseofulvin-producing fungi was carried out using the following culture media: potato dextrose broth KASVI^®^ (PDB); Potato dextrose agar KASVI^®^ (PDA); Czapek broth (CZK) (HIMEDIA^®^); Malt extract peptone broth (MEP): malt extract KASVI^®^ (30 g·L^−1^) and bacteriological peptone HIMEDIA^®^ (3 g·L^−1^); Wickerham medium modified (WKM) dextrose (10 g·L^−1^), malt extract KASVI^®^ (3 g·L^−1^), bacteriological peptone HIMEDIA^®^ (5 g·L^−1^), yeast extract (3 g·L^−1^) and micronutrient solution (10 mL.L^−1^) with FeSO_4_.7H_2_O (0.1 g), MnSO_4_.H_2_O (0.1 g), CuCl_2_.2H_2_O (0.002 g), CaCl_2_ (0.01 g), H_3_BO_3_ (0.0056 g), (NH_4_)_6_Mo_2_O_24_.4H_2_O (0.0019 g), ZnSO_4_.7H_2_O (0.02 g)); and rice medium (R) (50 g of rice added to an Erlenmeyer flask with 60 mL of water). All the culture media were autoclaved at 121 °C for 20 min before inoculation.

For liquid cultures, a two-stage fermentation procedure was employed. In the first step, for each culture medium, 10 fragments from previous PDA cultures of each strain were incubated at 28 °C for 7 days on a rotatory shaker (150 RPM) in 125 mL Erlenmeyer flasks with 40 mL of a respective culture medium described above. In the second step, the entire contents of each fermentation from the first step was added to Erlenmeyer flasks (500 mL) with 120 mL of the same culture medium, which were incubated in static condition at 25 °C for 28 days. For PDA cultures, each strain was cultured in 90 mm plates with 10 mL of culture medium at 25 °C for 28 days. For the rice medium, 10 fragments from previous PDA cultures of each strain were added to Erlenmeyer flasks (500 mL) containing autoclaved rice. All strain–medium combinations resulted in 36 cultures. Blank samples were prepared using the same methodology without inoculation of fungi.

After the fermentation time, each liquid culture was filtered to separate the mycelium and broth. The liquid phases were extracted two times with ethyl acetate. Solid cultures (PDA and R) were directly extracted two times with ethyl acetate. All solutions were filtered and, subsequently, dried by rotatory evaporation under reduced pressure at 40 °C furnishing the crude extracts. Each extract was prepared and analyzed according to previously described conditions (Section 3.2).

The LC–MS data files were converted from .d to .mzXML format using MSConvert software. Feature detection, grouping and alignment were performed using MZmine2 following the feature-based molecular networking documentation [41,52]. Data processing with MZmine2 generated two files, a CSV file (quantitative feature table) and an MGF file (MS/MS spectra for each feature), which were uploaded and used in feature-based molecular networking workflow in GNPS (http://gnps.ucsd.edu, accessed 20 March 2020). The precursor ion mass tolerance and fragment ion mass tolerance were both set to 0.05 Da. Molecular networks were generated with minimum of six matching peaks and cosine score of 0.7, and other parameters were maintained as default. Cytoscape 3.7.2 was used for visualization of the FBMN [53]. FBMN was selected in this work because it supports the elaboration of molecular networks integrated with chromatographic data. This workflow integrates chromatographic data (peak area and retention time) of a specific compound (feature) to its MS2 spectrum, enabling isomer differentiation and visualization of abundance and retention time of specific compounds into the network context [41].

### 3.4. Isolation, Identification, and Quantification of Griseofulvin and 7-Dechlorogriseofulvin

Extracts containing griseofulvin and 7-dechlorogriseofulvin were grouped and purified by semi-preparative HPLC. The sample was solubilized with MeOH (concentration of 10 mg·mL^−1^) and a sample injection volume of 40 µL was used in each chromatographic run. The chromatographic method employed was constituted by a gradient of mixtures of solvents A (0.1% acetic acid in H_2_O) and B (acetonitrile) of: 0–5 min (10–25% B); 5–15 min (25–50% B); 15–17 min (50–60% B); 17–17.5 min (60–100% B); 17.5–20 min (100% B). Both isolated compounds were identified by comparing ^1^H NMR data (Table S1) with literature data [54].

For calibration curve elaboration, each compound was solubilized in HPLC-grade acetonitrile (1 mg·mL^−1^) and filtered using a 0.45 µm syringe filter. The obtained solutions were diluted in a concentration range of 1–200 µg·mL^−1^, resulting in nine standard solutions of each compound. These samples were analyzed in triplicate by HPLC–DAD-UV, with an injection volume of 3 µL. The column temperature was set at 40 °C and the chromatographic method was constituted by a gradient of mixtures of solvents A (0.1% acetic acid in H_2_O) and B (acetonitrile) of: 0–5 min (10–25% B); 5–15 min (25–50% B); 15–25 min (50–100% B); 25–30 min (100% B); 30–35 min (100–10% A). The average peak areas vs. the concentration of each analyte were used to construct the calibration curve for each compound (Figures S3 and S4). The limits of detection (LOD) and quantification (LOQ) were calculated respectively for griseofulvin and 7-dechlorogriseofulvin as 0.17 µg·mL^−1^ and 0.81 µg·mL^−1^, and 0.24 µg·mL^−1^ and 0.76 µg·mL^−1^.

In order to quantify griseofulvin and 7-dechlorogriseofulvin the extracts from the OSMAC approach were solubilized in HPLC-grade acetonitrile (1 mg·mL^−1^) and filtered using a 0.45 µm syringe filter. The resulting samples were analyzed in triplicate by HPLC–DAD-UV with the same parameters used to elaborate the calibration curve. The integration of peak areas of both compounds detected in each extract were submitted to statistical analysis (one-way ANOVA and Tukey HSD) to determine the statistically significant differences (*p* = 0.05) in their production between extracts. To verify the difference of the peak areas between extracts, a Tukey HSD (honestly significance difference) test was applied. All statistical analysis and curve plots were elaborated using OriginLab 9.0 software. Statistically significant differences were assigned a letter from “a” to “e” for griseofulvin and from “a*” to “c*” for 7-dechlorogriseofulvin.

### 3.5. Cytotoxicity Assay

The cytotoxic activity of crude extracts obtained from OSMAC approach was evaluated against the human colon adenocarcinoma cell line (HCT-116, ATCC^®^ CCL-247™). The extracts were tested at a concentration of 50 µg·mL^−1^ using the MTT assay [55] according to an assay described in literature [56,57]. HCT-116 cells were cultured in RPMI 1640 medium and supplemented with 10% fetal bovine serum and 1% antibiotic. Cells were diluted to 5 × 10^4^ cells.mL^−1^ and plated in 96-well plates. Extracts previously solubilized in DMSO were added to each well, and the cells were exposed for 72 h. Negative and positive controls received DMSO and doxorubicin, respectively. Three hours before ending the incubation time, 0.5 mg·mL^−1^ of 3-(4,5-dimethylthiazol-2-yl)-2,5-diphenyl-2H- tetrazolium bromide (MTT) was added to each well and removed 3 h later. Formazan, a purple precipitate, was solubilized in DMSO and the absorbance measured at 595 nm using a multiple reader (Thermo Fisher Scientific, Waltham, MA, USA). Single-dose MTT data were transformed to a percentage of inhibition of growth after normalization with positive (100% of inhibition) and negative (0% of inhibition) controls. Extracts were considered cytotoxic when inhibiting over 75% of the cell growth at 50 µg mL^−1^.

## 4. Conclusions

Three endophytic fungi isolated from *Moquiniastrum polymorphum* (Asteraceae) were cultivated using the “one strain many compounds” (OSMAC) strategy and analyzed by feature-based molecular networking (FBMN) generated in the GNPS platform using HPLC–MS/MS data. To evaluate the efficiency of the OSMAC approach allied with FBMN analysis in the prospection of compounds of biotechnological interest, griseofulvin and 7-dechlorogriseofulvin were isolated and quantified using HPLC. The relative percentage of compounds allowed the inference of the best strain–medium combinations that maximize their productions. Therefore, the MEP and WKM culture media, respectively composed of maltose and glucose as carbon sources and both composed of peptone as a nitrogen source, produced the highest concentrations of griseofulvin and 7-dechlorogriseofulvin. Although the concentrations of griseofulvin achieved in this study are still lower than those observed in commercial strains, no quantification data of 7-dechlorogriseofulvin was available in literature. This approach allowed the observation of differences in medium-specific and strain-specific production of griseofulvin derivatives. In addition, cytotoxic activity variations were observed in most of the extracts. The results showed the necessity of further studies to improve the production of griseofulvin and derivatives by the selected strains.

## Figures and Tables

**Table 1:** Percentage (*w*/*w*) of griseofulvin and 7-dechlorogriseofulvin in all extracts obtained from fungi strain growth on different culture media.

	% of Griseofulvin ^2^			% of 7-Dechlorogriseofulvin ^2^		
Culture Media ^1^	611	620	658	611	620	658
CZK	3.80	0.80	1.30	1.02	0.29	0.55
MEP	7.24	7.61	3.63	2.94	1.78	0.63
PDB	0.0	0.0	0.0	0.0	0.0	0.0
PDA	0.0	7.03	0.52	0.0	0.37	0.09
R	1.34	2.25	0.26	0.58	0.28	0.45
WKM	0.37	7.74	3.13	1.61	4.54	0.61

^1^ CZK = Czapek broth; MEP = malt extract peptone broth; PDB = potato dextrose broth; PDA = potato dextrose agar; R = rice; WKM = Wickerham modified broth. ^2^ 611: *Phomopsis* sp.; 620: *Hypoxylon* sp.; 658: *Aspergillus* sp.

**Table 2:** Percentage mean of inhibition of proliferation of human colon carcinoma cell line (HCT-116) from the extracts obtained of fungi strains growth on different culture media ^1^.

	Crude Extracts Obtained from Culture Media ^2^					
Strains ^3^	CZK	MEP	PDB	PDA	R	WKM
611	43.5 ± 8.3	65.8 ± 2.6	58.7 ± 4.1	8.6 ± 11.0	62.8 ± 3.0	62.3 ± 1.4
620	36.3 ± 5.9	68.7 ± 0.7	61.1 ± 3.5	44.8 ± 7.2	71.4 ± 5.0	30.6 ± 2.5
658	48.2 ± 7.9	63.4 ± 4.3	59.6 ± 5.5	62.0 ± 5.4	64.4 ± 4.9	53.7 ± 6.1

^1^ % GI ± SEM = % inhibition of cell proliferation ± standard error of the mean. ^2^ CZK = Czapek broth; MEP= malt extract peptone broth; PDB = potato dextrose broth; PDA = potato dextrose agar; R = rice; WKM = Wickerham modified broth. ^3^ 611: *Phomopsis* sp.; 620: *Hypoxylon* sp.; 658: *Aspergillus* sp.

## Data Availability

The original .d files are available with corresponding author.

## Supplementary Materials

The following are available online, Figure S1: BPC, TIC and EIC chromatograms obtained from the PDA crude extracts of the three strains MPO611, MPO620 and MPO658. Figure S2: Feature-based molecular network of the 18 extracts from OSMAC approach. Each cluster is formed by interconnected group of nodes (colored circles). Figure S3: Calibration curve of griseofulvin obtained from HPLC analysis. Figure S4: Calibration curve of 7-dechlorogriseofulvin obtained from HPLC analysis. Table S1: ^1^H NMR data of griseofulvin and 7-dechlorogriseofulvin. Table S2: Quantification of griseofulvin and 7-dechlorogriseofulvin in crude extracts of each producing strain.
