# Supplementary material for: OSMAC Strategy Integrated with Molecular Networking for Accessing Griseofulvin Derivatives from Endophytic Fungi of Moquiniastrum polymorphum (Asteraceae)
Source: Molecules. 2021 Dec 2;26(23):7316. doi: 10.3390/molecules26237316 (PMC8658887; doi:10.3390/molecules26237316)
Supplement: Supplementary file 1 [file molecules-26-07316-s001.zip › molecules-1462010-supplementary.pdf]

molecules

OSMAC Strategy Integrated with Molecular Networking for Accessing Secondary Metabolites from Endophytic Fungi of *Moquiniastrum polymorphum* (Asteraceae)

Victor F. Farinella [1], Eunizinis S. Kawafune [1], Marcelo M. P. Tangerina [1], Helori V. Domingos [2], Leticia V. Costa-Lotufo [2] and Marcelo J. P. Ferreira [1,*]

[1] Departamento de Botânica, Instituto de Biociências, Universidade de São Paulo, São Paulo 05508-090, SP, Brazil
[2] Departamento de Farmacologia, Instituto de Ciências Biomédicas, Universidade de São Paulo, São Paulo 05508-090, SP, Brazil.
* Correspondence: marcelopena@ib.usp.br; Tel.: +55-11-3091-7546

Supplementary material

MPO611 – *Phomopsis* sp.

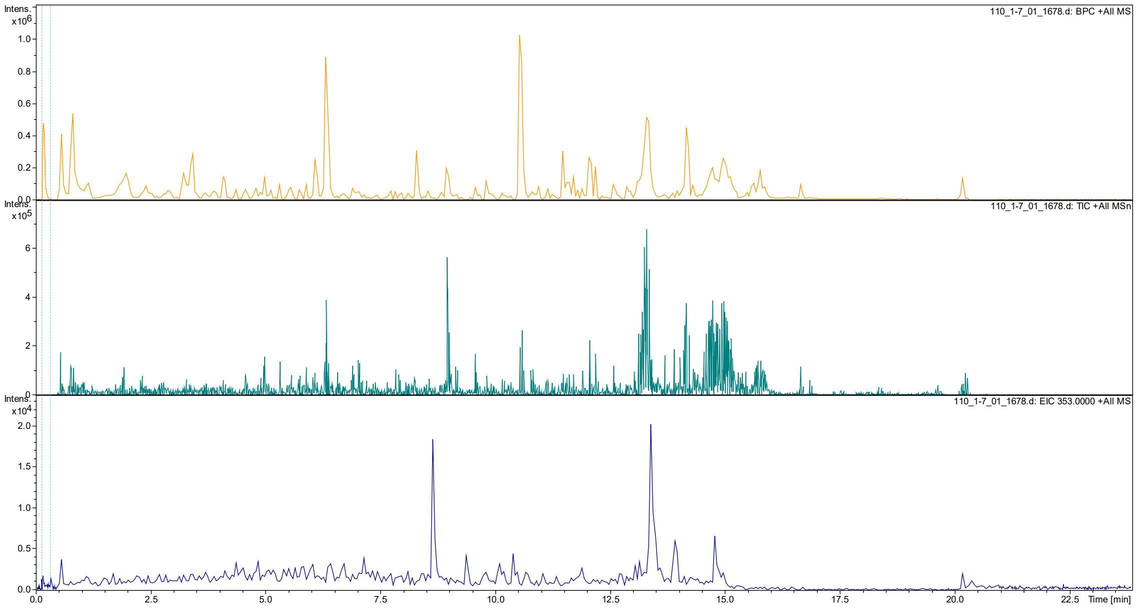

MPO620 – *Hypoxylon* sp.

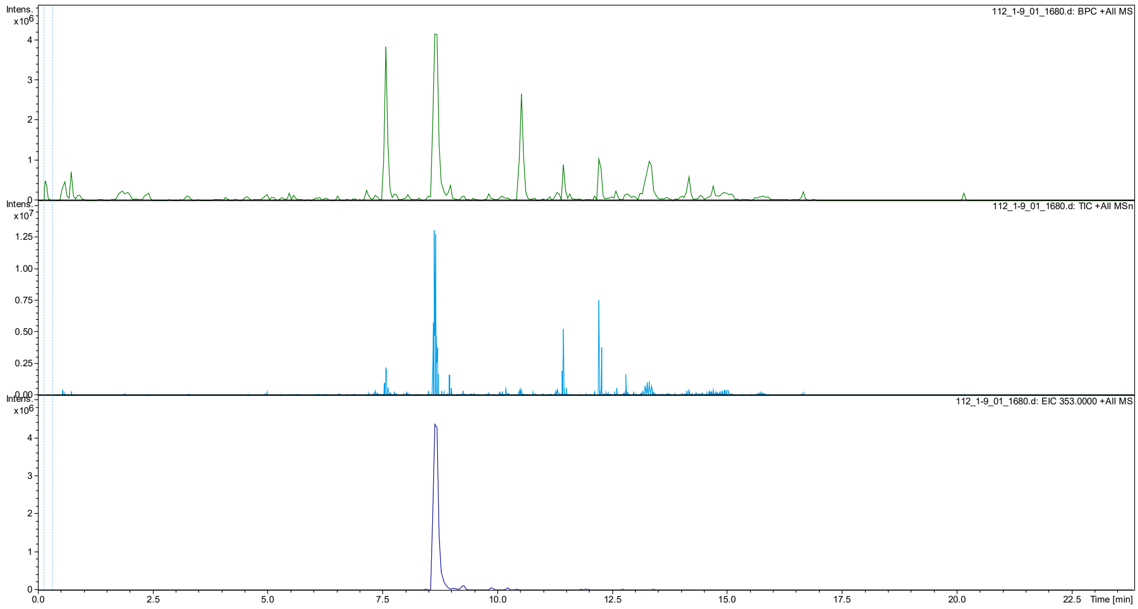

MPO658 – *Aspergillus* sp.

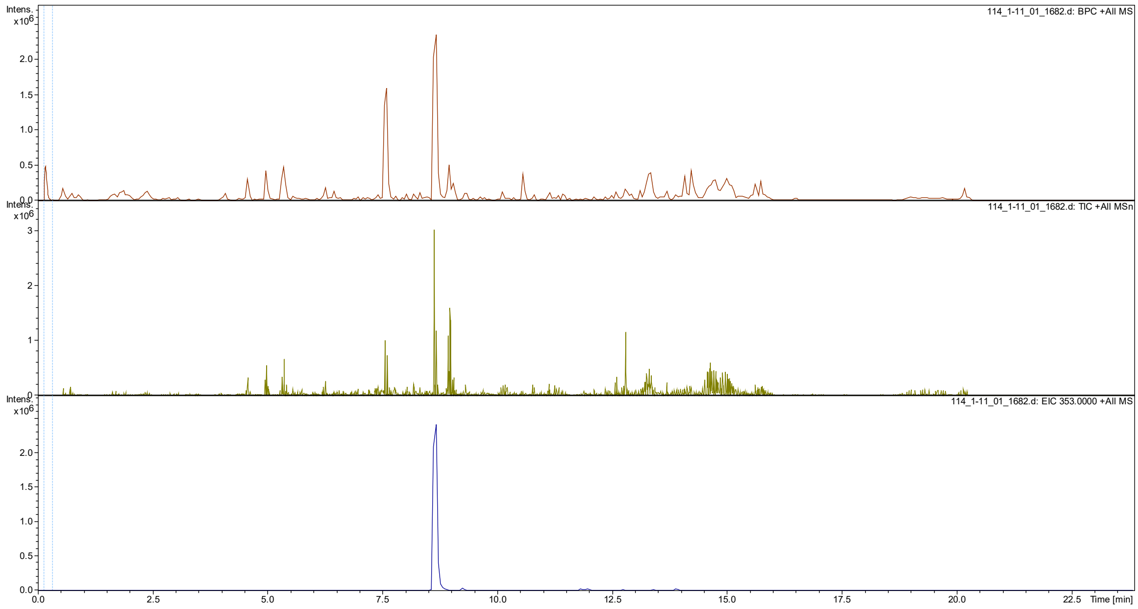

Figure S1. BPC, TIC and EIC chromatograms obtained from the PDA crude extracts of the three strains MPO611, MPO620 and MPO658.

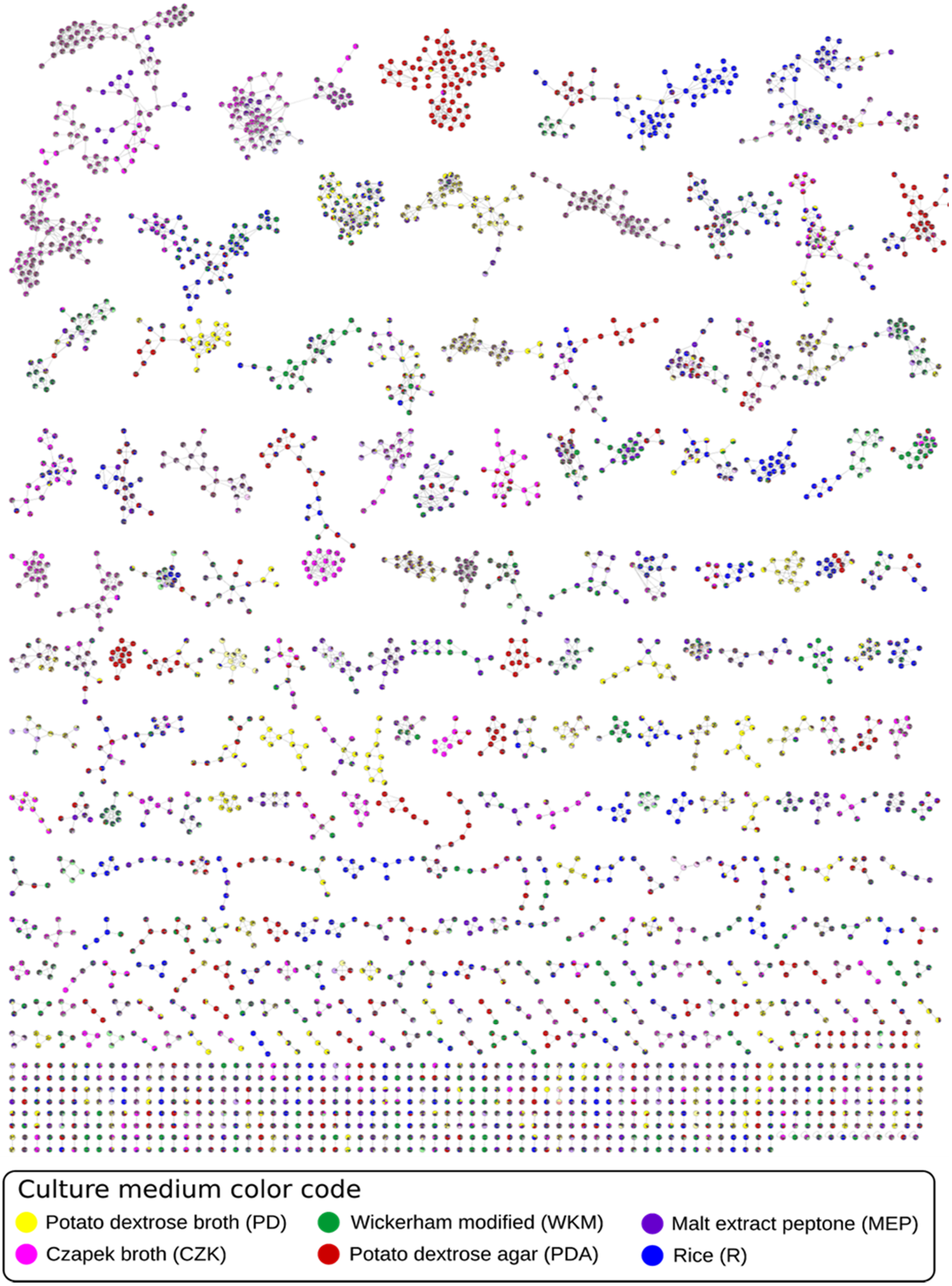

Figure S2. Feature-based molecular network of the 36 extracts from OSMAC approach. Each cluster is formed by interconnected group of nodes (colored circles).

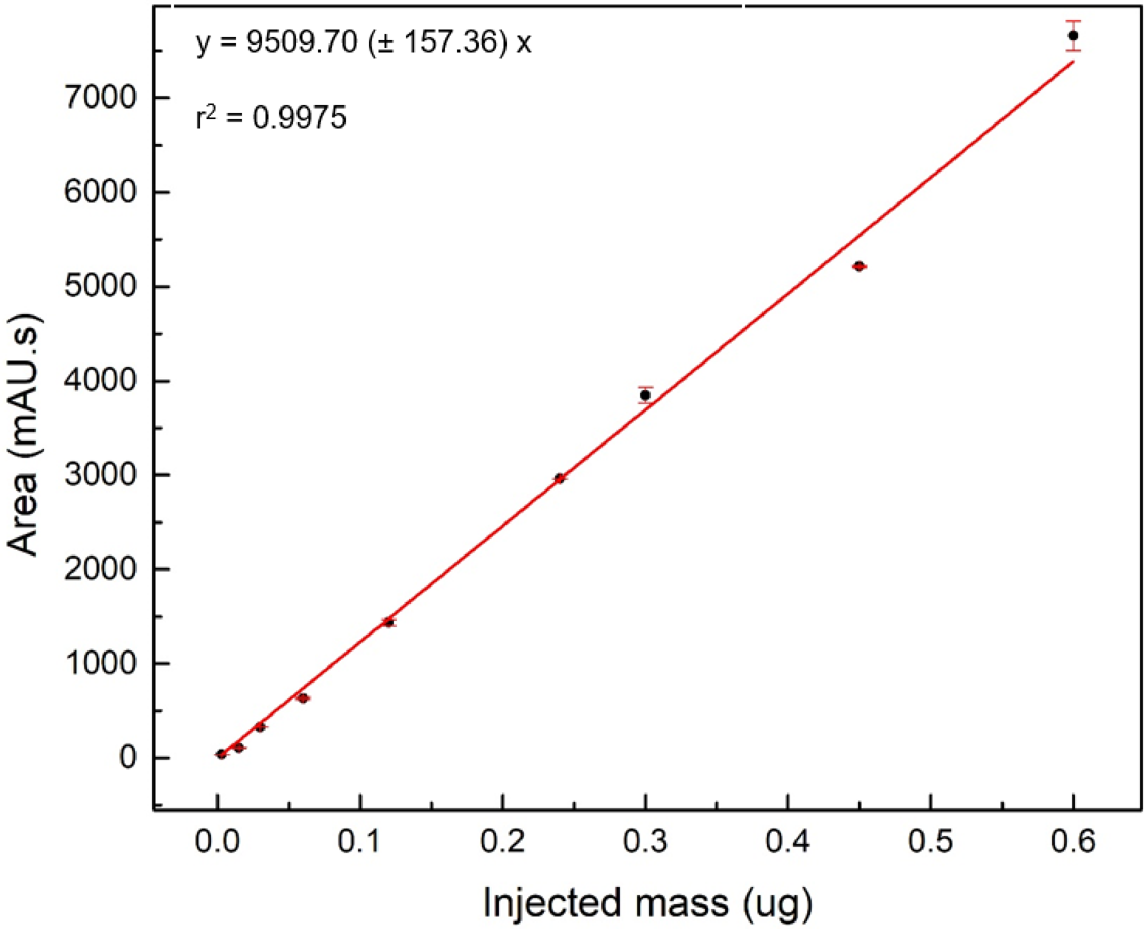

Figure S3. Calibration curve of griseofulvin obtained from HPLC analysis.

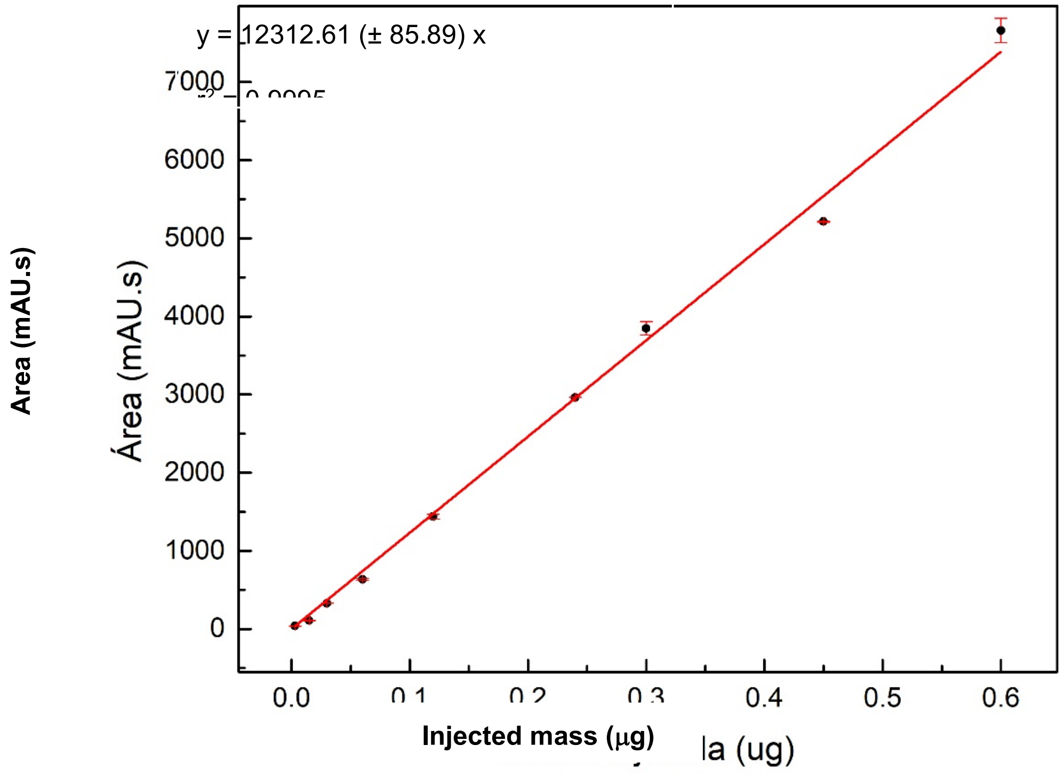

Figure S4. Calibration curve of 7-dechlorogriseofulvin obtained from HPLC analysis.

Table S1. ^{1}H NMR data (δ, multiplicity and *J* in Hz) of griseofulvin and 7-dechlorogriseofulvin.

Position	Griseofulvin	7-Dechlorogriseofulvin
5	6.08 (s)	6.17 (d, 1.5)
7	-	5.98 (d, 1.5)
3′	5.49 (s)	5.47 (s)
5′	2.39 (dd, 16.3; 5.0)<br>2.98 (dd, 16.3; 13.0)	2.32 (dd, 17.0; 5.0)<br>2.97 (dd, 17.0; 13.0)
6′	2.66 (m)	2.67 (m)
4-OMe	3.98 (s)	3.82 (s)
6-OMe	3.93 (s)	3.81 (s)
2′-OMe	3.58 (s)	3.56 (s)
6′-Me	0.90 (d, 7.0)	0.89 (d, 6.8)

Table S2. Quantification of griseofulvin and 7-dechlorogriseofulvin in crude extracts of each producing strain.

Culture media	Griseofulvin			7-Dechlorogriseofulvin		
	Area (mAU.s)	Mass (µg)	Concentration ($mg.L^{-1}$)	Area (mAU.s)	Mass (µg)	Concentration ($mg.L^{-1}$)
611-CZK	1082.75 ± 24.89 (c)	0.114 ± 0.003	0.40	378.70 ± 8.57	0.031 ± 0.001	0.11
611-MEP	2066.04 ± 6.05 (b)	0.217 ± 0.004	10.73	1094.00 ± 3.21	0.089 ± 0.001	4.39
611-PD	0.00 ± 0.00	0.000 ± 0.000	0.00	0.00 ± 0.00	0.000 ± 0.000	0.00
611-PDA	0.00 ± 0.00	0.000 ± 0.000	--	0.00 ± 0.00	0.000 ± 0.000	--
611-R	381.91 ± 11.22 (d)	0.040 ± 0.001	--	213.75 ± 3.38 (a*)	0.017 ± 0.000	--
611-WKM	106.45 ± 1.83 (e)	0.011 ± 0.000	0.87	597.42 ± 7.90	0.049 ± 0.001	3.79
620-CZK	226.89 ± 4.98	0.024 ± 0.001	0.07	109.37 ± 3.58 (c*)	0.009 ± 0.000	0.03
620-MEP	2170.42 ± 44.03 (a)	0.228 ± 0.006	10.79	662.25 ± 13.19	0.054 ± 0.001	2.54
620-PD	0.00 ± 0.00	0.000 ± 0.000	0.00	0.00 ± 0.00	0.000 ± 0.000	0.00
620-PDA	2004.30 ± 33.57 (b)	0.211 ± 0.005	--	137.87 ± 2.98 (b*)	0.011 ± 0.000	0.07
620-R	642.50 ± 0.74	0.068 ± 0.001	--	105.08 ± 0.38 (c*)	0.009 ± 0.000	--
620-WKM	2206.86 ± 51.39 (a)	0.232 ± 0.007	15.76	1687.55 ± 40.22	0.137 ± 0.003	9.31
658-CZK	369.79 ± 2.50 (d)	0.039 ± 0.001	0.18	202.81 ± 5.52 (a*)	0.016 ± 0.000	0.08
658-MEP	1034.31 ± 1.86 (c)	0.109 ± 0.002	5.19	234.29 ± 0.77 (a*)	0.019 ± 0.000	0.91
658-PD	0.00 ± 0.00	0.000 ± 0.000	0.00	0.00 ± 0.00	0.000 ± 0.000	0.00
658-PDA	148.51 ± 0.36 (e)	0.016 ± 0.000	--	34.74 ± 0.21	0.003 ± 0.000	--
658-R	72.98 ± 3.77 (e)	0.008 ± 0.000	--	168.59 ± 0.57 (b*)	0.014 ± 0.000	--
658-WKM	893.05 ± 5.96	0.094 ± 0.002	3.52	226.51 ± 1.79 (a*)	0.018 ± 0.000	0.69

Peak areas were submitted to ANOVA and a significative variance was observed for a p-value of p= 0.05. Tukey HSD test was applied to verify the peak area differences among extracts. Different letters (a) to (e) for griseofulvin and (a*) to (c*) for 7-dechlorogriseofulvin means statistic difference ($p<0.05$).
